# Polycaprolactone-Based, Porous CaCO_3_ and Ag Nanoparticle Modified Scaffolds as a SERS Platform With Molecule-Specific Adsorption

**DOI:** 10.3389/fchem.2019.00888

**Published:** 2020-01-10

**Authors:** Mariia Saveleva, Ekaterina Prikhozhdenko, Dmitry Gorin, Andre G. Skirtach, Alexey Yashchenok, Bogdan Parakhonskiy

**Affiliations:** ^1^Department of Biotechnology, Ghent University, Ghent, Belgium; ^2^Education and Research Institute of Nanostructures and Biosystems, Saratov State University, Saratov, Russia; ^3^Department of Nano- and Biomedical Technologies, Saratov State University, Saratov, Russia; ^4^Skoltech Center for Photonics and Quantum Materials, Skolkovo Institute of Science and Technology, Moscow, Russia

**Keywords:** SERS, Raman, calcium carbonate, silver nanoparticles, vaterite

## Abstract

Surface-enhanced Raman scattering (SERS) is a high-performance technique allowing detection of extremely low concentrations of analytes. For such applications, fibrous polymeric matrices decorated with plasmonic metal nanostructures can be used as flexible SERS substrates for analysis of analytes in many application. In this study, a three-dimensional SERS substrate consisting of a CaCO_3_-mineralized electrospun (ES) polycaprolactone (PCL) fibrous matrix decorated with silver (Ag) nanoparticles is developed. Such modification of the fibrous substrate allows achieving a significant increase of the SERS signal amplification. Functionalization of fibers by porous CaCO_3_ (vaterite) and Ag nanoparticles provides an effective approach of selective adsorption of biomolecules and their precise detection by SERS. This new SERS substrate represents a promising biosensor platform with selectivity to low and high molecular weight molecules.

**Graphical Abstract:**
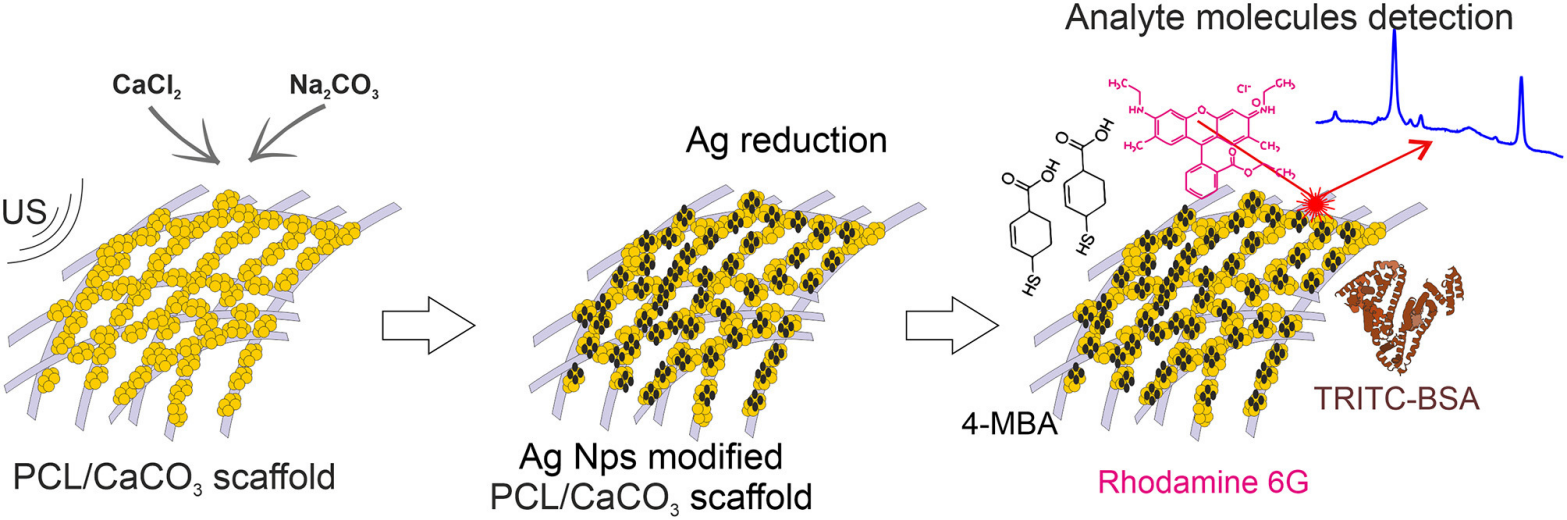
SERS platform for molecules with different ways of adsorption.

## Introduction

Raman spectroscopy is an analytical method, which provides information about molecular spectra and enables identification of chemical species. It is important in many areas, including biomedicine for early diagnosis and monitoring of diseases. However, Raman scattering signals are very weak. One of the most effective methods to enhance them is surface-enhanced Raman scattering (SERS), carried out by means of adsorption of silver, gold, etc. nanoparticles or nanostructures (Xu et al., 2014; Morrissey et al., 2015). In particular, strong amplification of electromagnetic fields of up to 10^11^ can be achieved within an interparticle junction (a “hot spot” area) or around them (Rycenga et al., 2012; Wang et al., 2014). Substantial efforts have been made for fabricating effective SERS platforms for environmental sensing (Bontempi et al., 2016), molecule detection by warped optical areas (Mao et al., 2018), ultrabright SERS sensors with embedded Raman tags (Jin et al., 2017), various types of dual-mode optical probes (Alvarez-Puebla et al., 2018), waveguide-based on-chip amplification (Raza et al., 2018), vertically aligned ZnO nanorod SERS substrates (Jue et al., 2019), fiber-based SERS probe (Kwak et al., 2019). Properties of nanoparticles and substrates play a crucial role in tuning SERS signal (Wi et al., 2012; Yashchenok et al., 2012; Kodiyath et al., 2013; Lin and Tang, 2015; López-Puente et al., 2015) as well as their distribution on the chip (Zalduendo et al., 2018). Furthermore, SERS has been applied in cell biology (Kneipp et al., 2006; Yashchenok et al., 2013; Krafft et al., 2017), where development of new super resolution methods should further contribute to localization of molecules. Porous colloidal particles of calcium carbonate or hydroxyapatite can be used for accumulation of analyte molecules in pores and thus increase the signal amplification, as it was demonstrated for adsorption of gold nanoparticles on their surface (Yashchenok et al., 2012). Furthermore, silver nanoparticles adsorbed onto calcium carbonate matrices have been demonstrated to substantially improve the SERS signal (Parakhonskiy et al., 2014; Kamyshinsky et al., 2019). Concentration of silver nanoparticles can be controlled either by adsorption conditions (Parakhonskiy et al., 2010) or direct synthesis (Hering et al., 2008; Parakhonskiy et al., 2010). The following functionalization them with magnetic nanoparticles will prove a possibility to move such SERS platforms under the magnetic field (Parakhonskiy et al., 2019). In addition, porous calcium carbonate particles have been shown to prolong the stability and detection capability of silver nanoparticles (Markina et al., 2018). An appropriate platform for deposition of such particles are scaffolds, for which electrospinning (ES) appears to be a very attractive fabrication technique (Prikhozhdenko et al., 2016; Saveleva et al., 2017; Chernozem et al., 2019).

Electrospinning is a simple and versatile technique capable of spinning fibers with diameters down to tens of nanometers. A variety of ES scaffolds with proper functionalization can be assembled for SERS. Indeed, ES nanofibers were shown to be an effective SERS substrate, because of a high surface to volume ratio and special optical properties achieved through the addition of metal or oxide nanoparticles (Severyukhina et al., 2015; Chen et al., 2017; Chamuah et al., 2018; Celebioglu et al., 2019; Restaino and White, 2019). The amplification of the signal, the impregnation of electrospun scaffolds with metal nanoparticles could enhance its optical, mechanical and morphological properties. The flexibility of the selection of polymers for ES allows producing electrospun scaffolds composed of biocompatible and environmentally friendly materials relevant for biomedical applications, including tissue engineering. Polycaprolactone (PCL) is attractive to ES, because it is non-toxic, chemically stable material available at a low cost. However, it is rather hydrophobic, which makes it challenging for biomedical applications. One solution to this problem is the surface functionalization of a scaffold to control its hydrophobic-hydrophilic (Duque Sánchez et al., 2016). The formation of hydrophilic inorganic porous bioceramic based coatings (for example, calcium phosphates CaP, calcium carbonates CaCO_3_, mesoporous silica) on a scaffold surface allows enhancing surface properties along with endowing of the additional functionality of drug delivery capability (Vallet-Regí et al., 2011; Savelyeva et al., 2017; Ivanov et al., 2019). The recently developed method of mineralization of the electrospun PCL scaffold with the CaCO_3_ porous ceramic microparticles allows obtaining homogeneous coatings in the vaterite phase overall fibrous electrospun scaffold by the *in situ* ultrasound-assisted CaCO_3_ syntheses at the surface of fibers (Savelyeva et al., 2017). Such biocompatible highly porous vaterite coating endows a scaffold with the following advantages: capability of encapsulating and targeted delivery of functional molecules and nanoparticles (including metal and metal oxide ones for SERS applications), and bioactivity (particularly, stimulating cell adhesion and increasing the surface hydrophilization; Saveleva et al., 2018; Ivanov et al., 2019).

Another aspect that appears to be important in SERS is the attachment of molecules—indeed, attached molecules have been reported to enable both stability and a high level of amplification (Wuytens et al., 2017).

In the present study, we design novel materials, which possess the functionalities of selective sorption of macro- and small- molecules along with amplification of their Raman signals for the detection. We hypothesized that electrospun PCL scaffolds sequentially modified with porous CaCO_3_ and silver Ag nanoparticles will provide selective sorption of the macro- and micromolecules and efficient amplification of the Raman signal by SERS. Ag nanoparticles were synthesized using two different methods: by *in-situ* reduction by the Tollen's reagents and by a similar reaction conducted in the presence of silver oxide, which is associated with hampering the SERS signal. Furthermore, such a comparison allows us to trace the influence of the oxidation on the SERS signal amplification.

## Materials and Methods

### Preparation of Mineralized Scaffolds PCL/CaCO_3_

The formation (electrospinning) of fibrous polymeric PCL scaffolds and subsequent mineralization of electrospun polymeric fibrous scaffold was performed by using approaches introduced previously in (Savelyeva et al., 2017).

### Modification of Scaffolds With Ag

Using clear Tollen's reagent: the 1 × 1 cm sample of scaffolds (PCL and mineralized PCL/CaCO_3_) were placed in 5 mL of Tollen's reagent [a mixture of 0.5 M AgNO_3_ (Sigma-Aldrich, Germany) and 0.5 M NH_4_OH (ChemReactiv, Russia) in a volume ratio is 1:1] for 10 min for adsorption of Tollen's complex on the surface of scaffold. After this, the scaffold was placed in a 40% solution of D-glucose for 10 min, then was washed with deionized water and dried at 40°C for 20 min.

Using Tollen's reagent containing 50% of silver oxide: the 1 × 1 cm sample of scaffolds (PCL and mineralized PCL/CaCO_3_) were placed in 5 mL of Tollen's reagent (0.5 M AgNO_3_: 0.5 M NH_4_OH in volume ratio 2:1) for 10 min for adsorption of silver oxides from solution on the surface of the scaffold. After this, the scaffold was placed in a 40% solution of D-glucose for 10 min, then was washed with deionized water and dried at 40°C for 20 min.

### The Adsorption of the Low and High Molecular Weight Molecuels

A solution of tetramethylrhodamine-bovine serum albumin (TRITC-BSA, 0.5 mg/ml, Sigma-Aldrich) was used as source as macromolecular with relatively high molecular weight (Mw = 70 kDA). A solution of Rhodamine 6G (Rh6G, 0.5 mg/ml, Sigma- Altrich) was used as low molecular weight substance (Mw = 479 Da) for loading and Raman studies. For confocal studies a solution of photosensitizer “Photosens”® (Russia) (Phs), representing a mixture of sulfonated aluminum phthalocyanines with various degrees of sulfonation (0.5 mg/mL), was used as a source of macromolecules.

For Raman and loading efficiency studies scaffolds with various modifications were immersed for 5 min in the solution of TRITC-BSA with initial concentration (Cini) 0.5 mg/ml in the solution with Rh6G with initial concentration (Cini = 0.5 mg/ml). After this the concentration of molecules in solution (Csn) were analyzed via UV-Vis spectrophotometry at appropriate wavelengths (567 nm for TRITC-BSA and 567 nm for Rh6G), and the Loading Efficiency (LE) was calculated using the following equation:

(1) $$\text{LE}=(\text{Cini–Csn}){\text{/Cini}}^{*}\text{100}\%$$

The mixture of TRITC-BSA and Phs was prepared in a 1:1 ratio (by volume) with initial concentrations: Cini(Phs) = 05 mg/ml and Cini(TRITC-BSA) = 0.5 mg/ml. The scaffold was immersed in the mixture and fixed in such position for 5 min for adsorption of molecules. The concentration of the molecules in solution was measured via UV-vis spectrophotometry (UVIKON XL Secomam), in an appropriate wavelength (567 for TRITC-BSA and 670 for Photosens). Loading efficiency was estimated via Equation (1). All LE ware calculated based on statistic of 5 samples.

### Characterization Methods

The scanning electron microscopy of scaffolds was performed in the manner as described in (Savelyeva et al., 2017). Raman and SERS measurements were carried out using Renishaw inVia (UK) spectrometer with a 785-nm laser irradiated through a 50× objective (Leica N PLAN 0.5 N.A.).

4-mercaptobenzoic acid (4-MBA, Sigma Aldrich, Germany) ethanol solutions (10^−3^, 10^−4^, and 10^−5^ M) were used as analyte. Prior to Raman and SERS measurements, all substrates (5 × 5 mm size) were put into 4-MBA solution (1 mL) for 20 min and then washed with ethanol. Raman spectra of 4-MBA (10^−3^ M) on PCL and mineralized PCL/CaCO_3_ were recorded with 10 mW laser power and were scanned for 5 s per spectrum. SERS maps (20 × 20 μm) of 4-MBA with concentrations 10^−3^, 10^−4^, and 10^−5^ M were recorded with 10 μW laser power and 5 s per spectrum. Enhancement factors (EF) were calculated according to the formula:

(2) $$\begin{array}{l} EF=\;\frac{I_{SERS}}{I_{Raman}}\cdot \frac{p_{Raman}}{p_{SERS}}\cdot \frac{c_{Raman}}{c_{SERS}}, \end{array}$$

where *I* —the intensity of the 1,580 cm^−1^ line for SERS spectra (I_*SERS*_) and that of the control analyte (I_*Raman*_); *p*—laser power for SERS spectra (pSERS) and control analyte (p_*Raman*_); c — the concentration (of 4-MBA) for SERS spectra (c_*SERS*_) and control analyte (c_*Raman*_). SERS data were represented as box-and-whiskers diagrams with the interquartile range 1.5 and no outliers plotted for SERS intensities.

TRITC-BSA or Rhodamine 6G water solution (0.5 mg/ml) was used as analyte which models high molecular weight molecule (protein) and low molecular weight molecule which was loaded like described above (Section The Adsorption of Low and High Weight Molecules). Raman spectra of analyte on PCL, mineralized PCL, functionalized with silver were recorded with laser power 0.5 mW 0.1 s per spectrum and with 50 mW 0.1 s per not functionalize with silver. SERS maps (20 × 20 μm). The enhancement factor was estimated via formula (2) were the intensity I—the intensity of the 1,513 cm^−1^ line.

## Results

**Figure 1:**
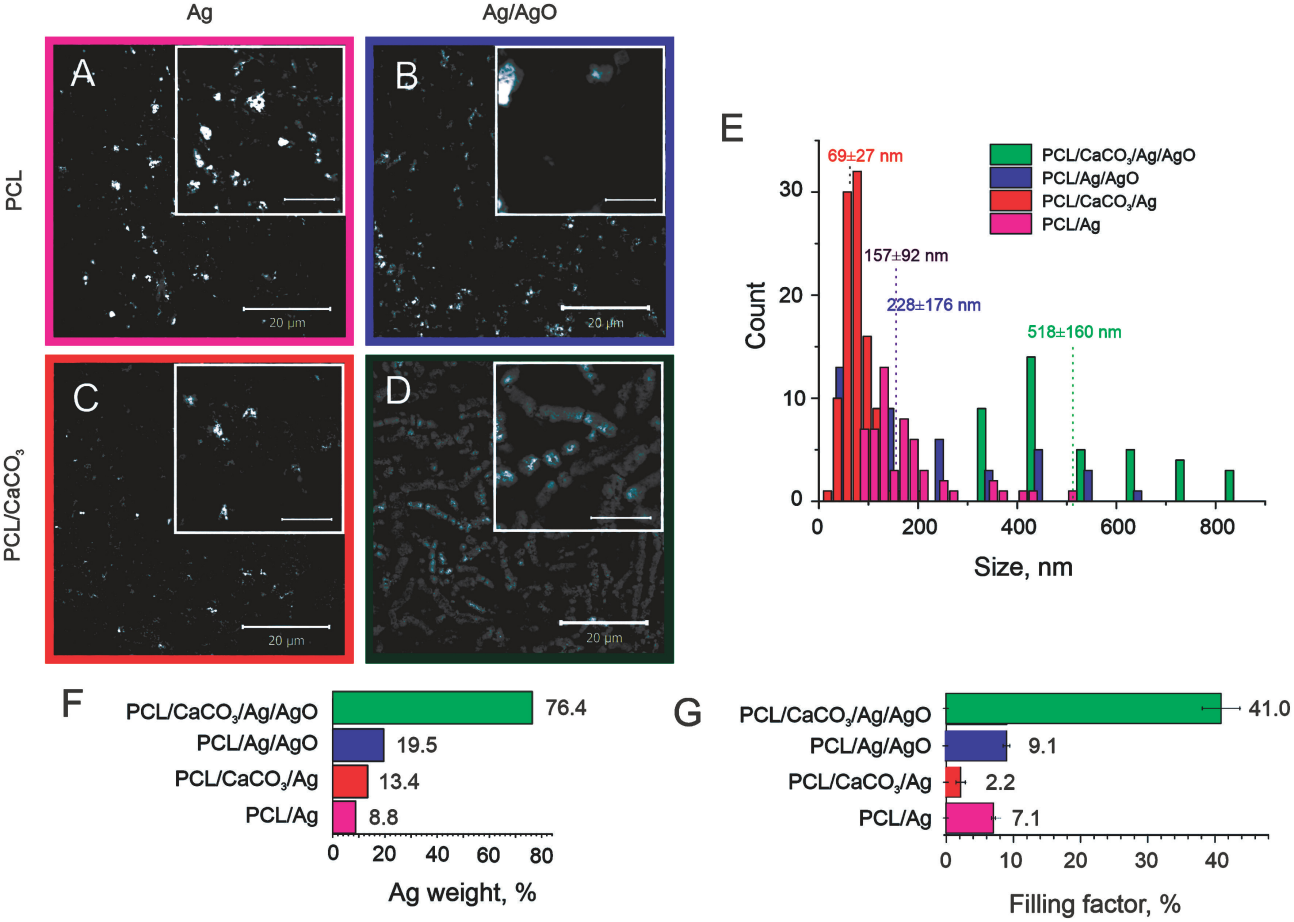
SEM images of the Ag reduction on non-mineralized PCL fibers **(A,B)** and mineralized PCL-CaCO_3_ scaffolds **(C,D)** after Ag reduction from the clear Tollen's reagent **(A,C)** and the reagent containing the Tollen's reagent with 50% of silver oxides **(B,D)**. The scale bars in inset images are 5 μm. **(E)** The size distribution of silver nanoparticles adsorbed on various scaffolds. **(F)** The silver content (weight percent) obtained via EDX analysis. **(G)** The filling factor of silver nanoparticles in various PCL scaffolds.

The Ag nanoparticles (AgNP) were synthesized by two methods: (1) from the clear Tollens reagent (produced with and denoted as Ag) and (2) the Tollens reagent mixed with silver oxide (produced with and denoted as Ag/AgO). Subsequently, their adsorption has been studied on scaffolds possessing porous CaCO_3_ (and referred to as CaCO_3_ mineralized) and those without CaCO_3_ (referred to as non-mineralized) scaffolds. That allows to investigate the influence of CaCO_3_ mineralization of PCL scaffolds on adsorption of Ag nanoparticles synthesized by the above mentioned two methods, Figure 1. This study is relevant, because the concentration, sizes, and the aggregation state of Ag nanoparticles determine the strength of the SERS amplification.

SEM images demonstrating adsorption of Ag and Ag/AgO on PCL scaffolds are shown in Figures 1A,B, respectively. Functionalization of non-woven PCL nanofibers was also carried out with CaCO_3_. SEM images of the morphology of PCL scaffolds mineralized with CaCO_3_ after subsequent reduction of Ag and Ag/AgO are shown in Figures 1C,D, respectively. Aggregation of nanoparticles leads to a higher SERS amplification; therefore, the aggregation state of nanoparticles is compared in the scaffolds before, Figure 1A (Ag on PCL), and after, Figure 1C (Ag on PCL/CaCO_3_), CaCO_3_ deposition. It can be seen from these images that in the former case a more agglomerated pattern of Ag particles is observed—calcium carbonate, thus, has not promoted the aggregate formation. CaCO_3_ affects adsorption of Ag/AgO, which is found to be higher (in weight percent) on PCL/CaCO_3_ scaffolds than that on PCL, Figure 1D.

We further present data on the sizes of nanoparticles (Figure 1E), the overall weight of adsorbed silver NP (Figure 1F) and their filling factor (Figure 1G), determined as the ratio of the sum of cross-sections of all individual nanoparticles to the total area (Skirtach et al., 2005). The average sizes of adsorbed Ag nanoparticles depend on the mineralization with CaCO_3_, in which case it is almost two times lower. Specifically, AgNP adsorbed from the Tollen's reagent were obtained in the size range of ~ 70 ± 30 nm for the mineralized scaffolds, while they were ~ 160 ± 90 nm for the non-mineralized scaffolds, Figure 1E. The size difference is thus by a factor of more than 2. In contrast to data obtained with the Tollen's reagent only (Ag), the size of AgNP obtained from the mixture of Tollen's agent and Ag/AgO the average size of AgNP is also higher by the factor of ~2, but in this case, the size of AgNP is ~520 ± 160 nm for mineralized with CaCO_3_ scaffolds versus 230 ± 180 nm for non-mineralized ones.

**Figure 2:**
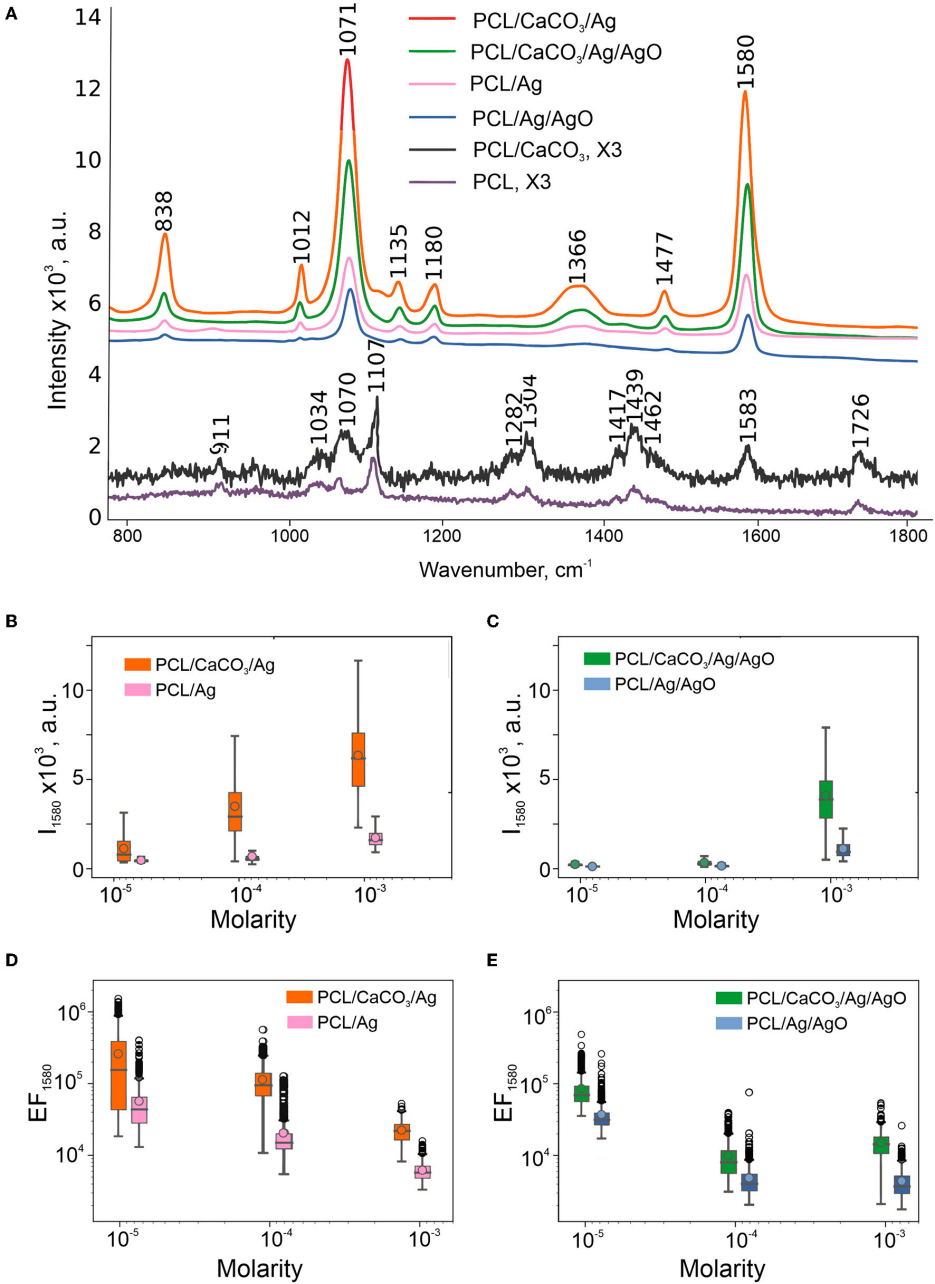
SERS performance of obtained scaffolds: Average spectra of 4-MBA (10^−3^ M) on SERS-substrates calculated from maps 20 × 20 μm and multiplied by 3 spectra on PCL, PCL/CaCO_3_ fibers **(A)**. Box-and-whiskers plots of intensity at 1,580 cm^−1^ of SERS substrates without **(B)** and with 50% of silver oxides **(C)**. Data on SERS substrates without CaCO_3_ are shown in the insets. Enhancement factors calculated from these intensities according to the formula in Section Characterization Methods are shown in the log scale **(D,E)**.

To define and match the SERS performance of different scaffolds, we used 4-mercaptobenzoic (4-MBA) acid as an analyte. 4-MBA has a high affinity to silver nanoparticles through the thiol group. But, there was no signal of 4-MBA (10^−3^ M) on PCL scaffolds without CaCO_3_ (Figure 2A, the violet and bottom line), while all of the present peaks correspond to PCL (Kotula et al., 2017). After the mineralization procedure, there was an additional peak at 1,583 cm^−1^ corresponding to the 4-MBA ν(C–C)_ring_ mode (Orendorff et al., 2005). This peak intensity was used as 4-MBA Raman intensity for further EF calculations. The average SERS scattering intensities and the EF at 1,580 cm^−1^ are shown in Figures 2B–E and in a Table S1.

To reveal the difference between specificity of molecule adsorption TRITC-BSA (Mw = 70 kDa) and Rh6G (Mw = 479 Da) as large and small molecule have been chosen. Both of these molecules have the same mechanism of adsorption–the physical sorption on the surface of the scaffold. It was found that the loading efficiency (LE) were much higher for Rh6G (40%) than for TRITC-BSA (24–30%). The presence of silver nanoparticles (AgNPs) on PCL scaffold increase large molecule sorption for non-mineralized scaffolds (PCL, PCL/Ag).

**Figure 3:**
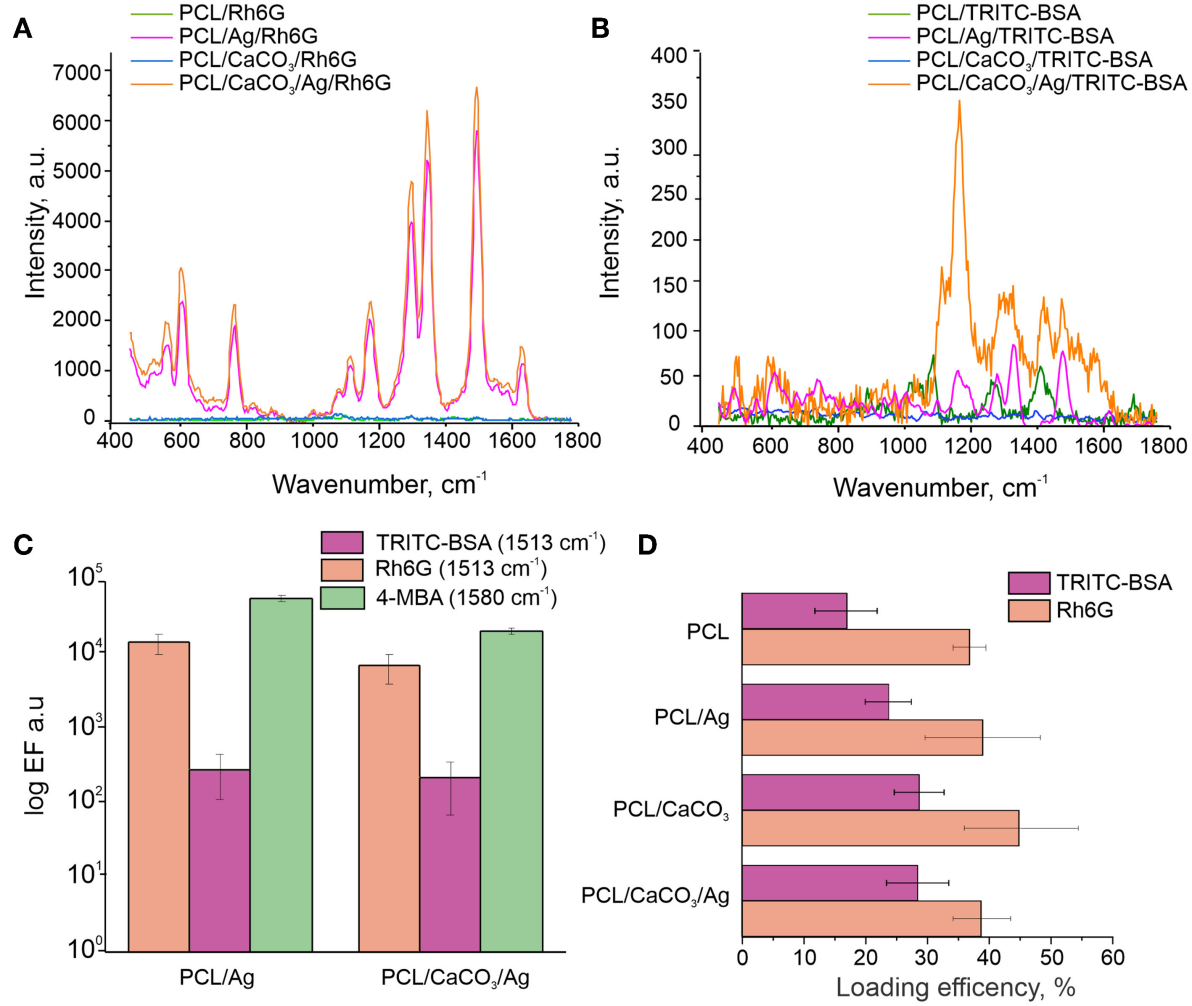
Raman spectra of the Rh6G **(A)** and TRITC-BSA **(B)** on various scaffolds. **(C)** Comparison of the enhancement factor of the C-C aromatic stretching peak (1,513 cm^−1^) for TRITC-BSA and Rh6G and at 1,580 cm^−1^ for 4-MBA in log scale. **(D)** The loading efficiency (LE) for TRITC-BSA (pink) and Rh6G (orange).

The SERS spectra revealed several peaks of Rh6G in the region between 500 cm^−1^ and 1,700 cm^−1^ (Figures 3A,B). The most intense peaks at 1,314, 1,363, 1,512, and 1,651 cm^−1^ are assigned to C-C aromatic stretching. The SERS spectra of TRITC-BSA displayed three peaks of BSA at 1,203, 1,453, and 1,654 cm^−1^, which are assigned to Amide III, CH_2_ bending and Amide I, respectively. Two peaks at 1,363 and 1,513 cm^−1^, representing a contribution from TRITC, are also detected for SERS spectra of TRITC-BSA. The enhancement factor (Figures 3C,D) of both analyzed molecules (TRITC-BSA and Rh6G) was estimated using the intensity at 1,513 cm^−1^ which corresponds to C-C aromatic stretching peak of Rh6G. Comparison of Enhancement factor showed that the scaffolds without AgNP demonstrate the Raman signal intensity <50, as with high laser power (50 mW) On the other hand, the samples modified with AgNP (PCL/Ag and PCL/CaCO_3_/Ag) demonstrate the SERS effect with the signal intensity of up to 10^5^ that for Rh6G and up to 500 for TRITC-BSA. Peculiarly, there is no significant difference (based on ANOVA tests) between signal amplification on modified and non-modified by calcium carbonate samples. Eventually, this means that macro roughness due to AgNP is a dominant factor for adsorption of small and large molecules regardless of the size of molecules.

**Figure 4:**
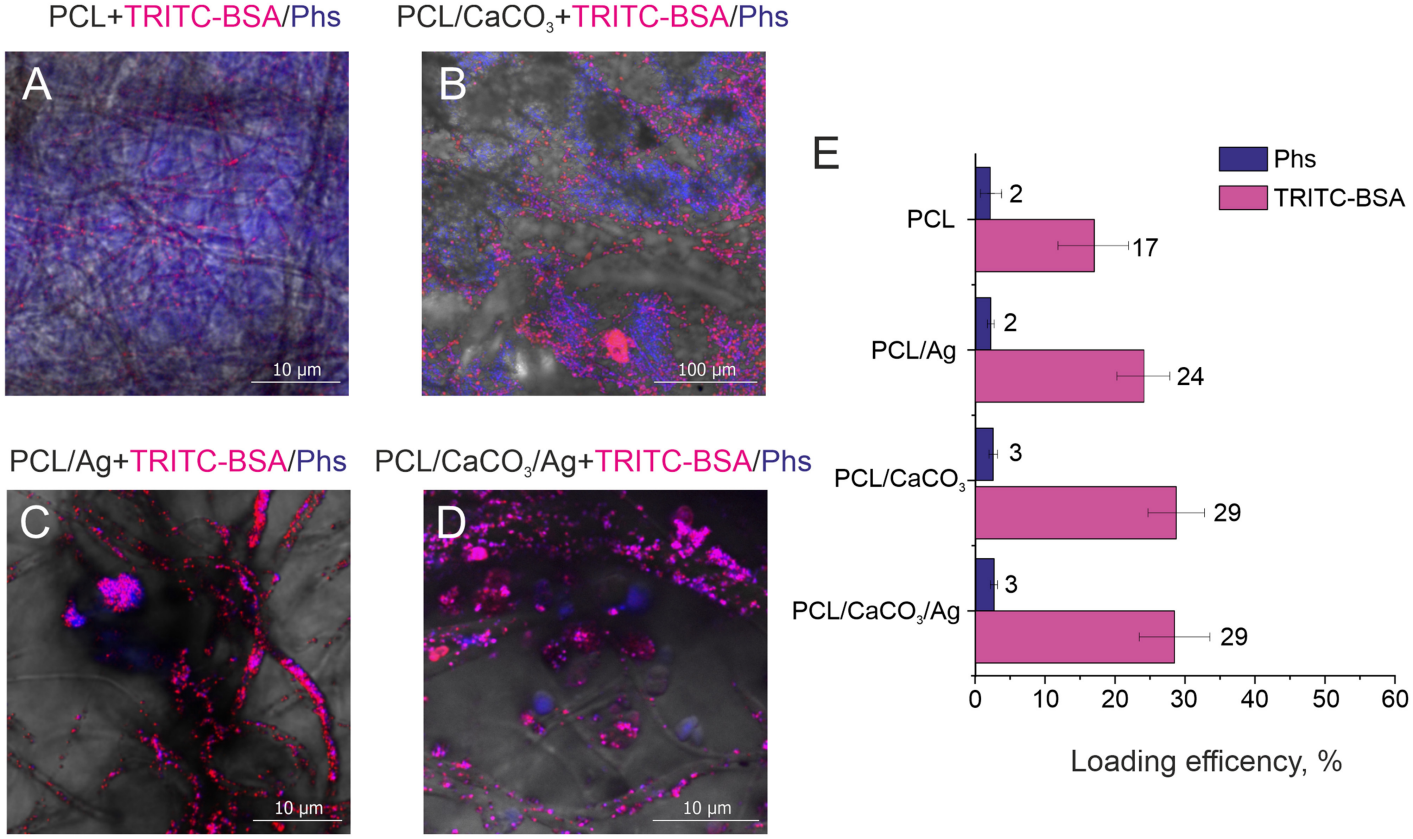
Confocal images of different scaffolds **(A)** polycaprolactone, **(B)** PCL/CaCO_3_. **(C)** PCL/Ag, and **(D)** PCL/CaCO_3_ matrix after Ag immersed in a mixture of Photosens and TRITC –BSA. **(E)** The loading efficiency for different scaffolds for Phs (blue) and TRITC-BSA (pink).

Ensuing study of the sorption process was carried out using fluorescence microscopy. Here, we have used TRITC-BSA as a large molecule, while Photosens (Phs) is used as a small molecule. The molecular weight of Phs is 574.9 Da, which is comparable to that of Rh6G (479.2 Da). However, Photosens has a strong fluorescence signal at a different wavelength (with the emission maximum at around 689 nm). Our results reveal that it is possible to recognize both of these molecules using confocal fluorescence microscopy (Figure 4).

Confocal microscopy images (Figures 4A–D) complemented by a same images split by different fluorescent channels (Figures S3–S6) demonstrate the distribution of small and large molecules in various scaffolds. Significant differences can be observed between mineralized and non-mineralized scaffolds, along with the obvious influence of the presence of Ag particles on the separation performance of molecules. In the case of the non-mineralized scaffold without CaCO_3_ and Ag, no separation and no characteristic aspects can be revealed from the image. Mineralized scaffold PCL/CaCO_3_ exhibits the conspicuous separation of Phs and TRITC-BSA pronounced in selective adsorption of small Phs molecules by vaterite coating and accumulation of TRITC-BSA macromolecules as aggregates at the surface of vaterite-coated fibers without adsorption. The decoration of scaffolds with Ag particles suppressed the adsorption of small molecules by vaterite due to Ag filling of adsorption sites of CaCO_3_. In this case, molecules distribution expressed in the form of molecules clusters and aggregates on the surface of the fibers—this idea confirmed by a study of the sorption molecules (Figure 4E). Small molecule better adsorbed on porous vaterite structure. Due to the method sensitivity for small molecule sorption has a higher deviation than for TRITC BSA. Vaterite has a significant increase in the loading capacity of TRITC-BSA as well, but silver nanoparticles don't provide any significant effect on the sorption efficiency.

Therefore, the modification of the scaffold by porous particles resulted in a significant (2 times) increase of the sorption of the large molecule and a slight increase of the sorption of a small molecule. The functionalization of the silver nanoparticles, which can provide the SERS amplification, but it doesn't significantly influence the loading properties of modified scaffolds.

## Discussion

The presence of a vaterite coating leads to both an increase of the hydrophilization of a surface and to an increase of the surface area and porosity, which influence the formation of Ag nanoparticles. Analysis of SEM images in combination with EDX data shows that the scaffolds coated with porous vaterite coating, while using the Tollens reagent without additional oxide let to the formation of smallest silver nanoparticles (~70 nm) (Figures 1A–D). It should be noted that the space confinement of the pores of calcium carbonate particles pose constrictions for AgNPs particle growth leading to their narrow size distribution. In the case of non-porous scaffolds, i.e., pure PCL, the Ag particles grow mostly in the solution followed with the adsorption on the PCL structure. Such an increase in a confined volume leads to the formation of much larger particles with sizes ~160 nm ± 90 nm. We note that the presence of silver oxide in the reaction mixture serves as a template for additional growth of the seeds for the Ag reduction. In this case, the particle size increases significantly up to 230 nm for PCL scaffolds and up to 520 nm for PCL/CaCO_3_. It can be noted that for PCL/CaCO_3_/Ag + 50% AgO and PCL/Ag + 50% AgO the filling factor as well as the Ag element content increase significantly. Moreover, in the case of vaterite-coated scaffolds, almost total coverage of the scaffolds with Ag is achieved.

Furthermore, the filling factor varies in the scaffolds from 2% till 40% and it is substantially higher for non-mineralized scaffolds (~7.1) in comparison to that for CaCO_3_ mineralized ones (~2.2) (Figure 1G). In the case of the Tollen's reagent containing 50% of silver oxide (Ag/AgO), the difference in Ag particle contents for not-mineralized and mineralized scaffolds can be observed from SEM images and EDX results (Figures 1A–D) together with Figures S1, S2. The Ag filling factor is higher by ~4 times for mineralized scaffolds in comparison with that for the non-mineralized scaffolds, Figure 1G. More specifically, the Ag content is 76.4 wt.% in the mineralized scaffolds, while it is only 19.5 wt.% in the case of the non-mineralized scaffolds. The X-ray element maps confirm the larger Ag content and the denser distribution of Ag particles in the mineralized scaffold in comparison with the non-mineralized scaffold in the case of using Ag/AgO nanoparticles.

Even though the Ag content (or in other words the filling factor) is much higher on fibers of scaffolds with AgO, the SERS EFs are observed to be higher on PCL/Ag and PCL/CaCO_3_/Ag without AgO (Figures 2B–E). Lower SERS EFs can be assigned to 2 factors: (1) the presence of silver oxide film resulting in a poor adsorption of the 4-MBA due to S atoms and carboxyl groups, and (2) plasmon damping on dielectric oxide films. For this reason, investigation of scaffolds with AgO is not further pursued.

We further investigate the SERS amplification comparing PCL, PCL/CaCO_3_, PCL/Ag, and PCL/CaCO_3_/Ag scaffolds. The following molecules have been used as analytes: (a) TRITC BSA (higher molecular weight molecule); (b) 4-MBA (small molecule, which is chemically cross-linked with the silver nanoparticles); and (c) Rh6G (small molecule, which is physically adsorbed to the surface). The discussion begins with scaffold adsorption of 4-MBA molecule—this process is referred to as chemisorption, i.e., in this case 4-MBA adsorption on the Ag surface via carboxyl groups and S atoms. The sorption efficiency depends on the filling factor of Ag nanoparticles and for PCL/Ag scaffolds the amount of the adsorbed molecules is 3 times higher than that for PCL/Ag/CaCO_3._ Furthermore, chemical adsorption of molecules is higher for mineralized PCL scaffolds (PCL/CaCO_3_ and PCL/CaCO_3_/Ag) in comparison with that for non-mineralized scaffolds (PCL, PCL/Ag), as demonstrated by the appearance of the peak in the Raman spectrum of chemically adsorbed 4-MBA of PCL/CaCO_3_ scaffold without silver nanoparticles (Figure 3A). At higher concentrations of 4-MBA, the Raman signal intensity is higher compared to that at a lower concentration, Figures 2B,C, but the EF of SERS has an inverse dependence on concentration. Indeed one can see in Figures 2D,E that the EF of SERS (calculated by Equation 1) at 10^−3^ M is about 10–15 times lower than that at 10^−3^ M of 4-MBA. This is presumably due to the saturation of the sorption of 4-MBA molecules on the scaffolds. The SERS signal on PCL/CaCO_3_/Ag substrates has the mean EF of 2.8 × 10^5^ with EF of 1.6 × 10^6^ in “hot spots.”

It is essential to note that even though the filling factor is higher for PCL/Ag (without CaCO_3_) than that for PCL/CaCO_3_/Ag, the SERS effect is stronger for the latter sample (PCL/CaCO_3_/Ag). It could be because of a higher amount of smaller nanoparticles (70 nm with CaCO_3_ vs. 160 nm without CaCO_3_, Figures 1A,C,E) provides a better distribution of Ag nanoparticles for SERS amplification. This is achieved by creating nanometer sized gaps between Ag nanoparticles, which essentially facilitate the SERS amplification.

On the other hand, the loading efficiency of molecules, which are physically adsorpbed on scaffold structures depends on the surface area and roughness of scaffolds as well as on the shape and size of to be analyzed molecules. For the following fluorescenct molecules: TRITC-BSA, Rh6G, Phs it is possible to directly measure their loading efficiency (Equation 1). First of all, we probe the spatial distribution of adsorbed small and large molecules on PCL and PCL/CaCO_3_ scaffolds functionalized with Ag nanoparticles. For this purpose, a pair of biomedically relevant molecules TRITC-BSA (a high-molecular weight protein BSA labeled with TRITC) and Photosens® (Phs) (photodynamic drug) molecules is chosen due to a non-overlapping nature of their fluorescent signals. Functionalization of PCL scaffolds with CaCO_3_ and AgNP is shown in our study to be useful for concentrating molecules. Indeed, Figure 4 shows images of PCL nanofibers after incubation in a solution containing these two molecules. It should be noted that the typical pore sizes of vaterite ranges from 30 to 90 nm, which limits incorporation of larger molecules, while smaller ones would more effectively penetrate inside the vaterite interior (Parakhonskiy et al., 2012). At the same time, large molecules would not penetrate inside the vaterite pores as efficiently as the small ones, and they would be adsorbed only at the external vaterite surface.

The scaffold modification by Ag has a significant influence on the adsorption of all molecules. In this regard and based on the confocal image analysis, it is observed that in the case of scaffolds without Ag small molecules penetrate inside the vaterite pores, while large molecules are adsorbed on the surface and are clustered in between the vaterite structures. In the case of Ag-decorated scaffolds, Ag nanoparticles fill the vaterite pores, blocking them and decreasing the sorption of small molecules. As a result, small molecules, Phs, are clustered similarly to large molecules (proteins in this case). The same effect is obtained for another pair of small and large molecules: Rh6G and TRITC-BSA, respectively (Figure 3D). The loading efficiency of the modified PCL scaffolds by calcium carbonate and/or AgNP provides a better sorption of large and small molecules than the non-modified scaffolds. The SERS signal amplification of TRITC-BSA is lower than that for either of the small molecules: 4-MBA and for Rh6G (Figure 3C). The scaffolds with AgNP provide amplification for Rh6G up 10^5^ which are comparable with the amplification (5*10^5^) of chemically bound 4-MBA, but in the case of physical sorption functionalization of the scaffold with the calcium carbonate don't give any advantage.

We further investigate SERS effect of the TRITC BSA induced by the C-C aromatic stretching of the rhodamine part of the molecule, because BSA has a low scattering cross section compared to that of rhodamine. For large molecule TRITC-BSA, the SERS amplification is not so significant, just 500 times with respect to the samples without AgNPs. It can occur due to three reasons: (1) smaller number of molecules providing C-C aromatic stretching because of: (a) an inefficient sorption of TRITC-BSA in comparison to that for Rh6G, and (b) the grafting ratio of TRITC on BSA is only 0.5 M per 1 M of BSA; (2) the presence of the BSA prevents an efficient contact of C-C atoms with Ag nanoparticles.

Nanostructures including electrospun scaffolds composed of such biocompatible materials as polycaprolactone, chitosan, polyurethane, polycaprolactone, etc., are identified here as an effective SERS amplification platform. Their modification, for example, by inorganic nanoparticles leading to the so-called hybrid (Saveleva et al., 2019) structures or scaffolds makes them a unique platform, where the porosity and the concentration or the filling factor of the metal nanoparticles can be controlled. Furthermore, their uniqueness lies in a possibility to adsorb molecules—a property, which has been identified as a very important factor enabling high and stable amplification (Wuytens et al., 2017). This principle, demonstrated in this work for porous materials in combination with their adsorption of molecules, resulted in significant amplification of the surface-enhanced Raman signals relevant for detection of a wide range of molecules.

## Conclusion and Perspectives

Polycaprolactone scaffolds, mineralized in this work by both calcium carbonate in the form of vaterite and Ag nanoparticles in the vaterite pores, are shown to be an effective SERS platform for dual- and molecularly selective detection. The SERS effect is provided by silver nanoparticles in a combination with a high specific adsorption facilitated by the porous structure of vaterite. The mineralization of the scaffolds by vaterite is found to play a key role in the silver nanoparticle formation, and it has led to a more homogeneous coverage of AgNP with the sizes ~70 nm. It is found in our studies that the SERS amplification for both TRITC-BSA and Rhodamine 6G strongly depends on the presence of Ag nanoparticles, because the latter provides not only the SERS amplification, but they also form a rough surface, on which molecules can attach better. The SERS amplification is found to be better for Rhodamine 6G (up to 10^5^) than for TRITC-BSA (up to 10^3^) assigned to a larger concentration of adsorbed Rhodamine 6G molecules and, most likely, their closer location to Ag nanoparticles. The SERS amplification signal of TRITC-BSA, monitored by the C-C aromatic stretching corresponding to the peak provided by TRITC functionalization, is worse than that for Rhodamine 6G. This is attributed to the presence of protein (BSA), which would prevent a close proximity of Rhodamine of TRITC-BSA to Ag nanoparticles. 4-MBA molecules, attached to scaffolds functionalized with Ag nanoparticles, have produced a good SERS amplification respect to the Rh6G and TRITC-BSA molecule. On the other hand, the amount of cross-linked 4-MBA is proportional to Ag nanoparticles, which directly give rise to the SERS signal amplification and which grow significantly better on Ag nanoparticles. It is also found in our studies that the presence of silver oxide in the reaction mixture significantly decreases the enhancement factor. Strong SERS magnification factors coupled with possibilities to probe molecular specific adsorption are characteristic features of these new scaffolds.

## Data Availability Statement

All datasets generated for this study are included in the article/Supplementary Material.

## Author Contributions

MS and EP contributed to writing and performed experiments, on which this work is based. DG, AY, BP, and AS have organized work and led some of research directions, on which this work is based.

### Conflict of Interest

The authors declare that the research was conducted in the absence of any commercial or financial relationships that could be construed as a potential conflict of interest.
