# Supplementary material for: Polycaprolactone-Based, Porous CaCO3 and Ag Nanoparticle Modified Scaffolds as a SERS Platform With Molecule-Specific Adsorption
Source: Front Chem. 2020 Jan 10;7:888. doi: 10.3389/fchem.2019.00888 (PMC6967418; doi:10.3389/fchem.2019.00888)
Supplement: Supplementary file 1 [file Table_1.pdf]

Polycaprolactone-based, porous $CaCO_3$ and Ag nanoparticle modified scaffolds as a SERS platform with molecule-specific adsorption

Mariia Saveleva[1,2^], Ekaterina Prikhozhdenko[2^], Dmitry Gorin[3], Andre G. Skirtach[1]*, Alexey Yashchenok[3]*, Bogdan Parakhonskiy[1]*

[1] Department of Biotechnology, Ghent University, 9000 Ghent, Belgium

[2] Department of Nano- and Biomedical Technologies, Saratov State University, 410026 Saratov, Russia

[3] Skoltech center of Photonics & Quantum Materials, Skolkovo Institute of Science and Technology, 143026 Moscow, Russia

*** Correspondence:**
Bogdan Parakhonskiy: bogdan.parakhonskiy@ugent.be

Alexey Yashchenok: a.yashchenok@skoltech.ru

Andre Skirtach: andre.skirtach@ugent.be

Keywords: SERS1, Raman2, calcium carbonate3, silver nanoparticles4, vaterite5.

Table S1. Average value of calculated enhancement factor (mean) and max value from 'hot spots' (max) obtained from the various concentration of 4-MBA adsorbed on various scaffolds

	Mean EF and max EF of 4-MBA							
Concentration of 4-MBA	PCL fibers				PCL/$CaCO_3$			
	Ag		Ag/AgO		Ag		Ag/AgO	
	mean	max	mean	max	mean	max	mean	max
10^{-3} M	5.8×10^3	1.5×10^4	4.0×10^3	2.4×10^4	2.1×10^4	5.0×10^5	1.4×10^4	4.9×10^5
10^{-4} M	2.0×10^4	1.3×10^5	5.7×10^3	8.9×10^4	1.1×10^5	5.6×10^5	1.2×10^4	4.6×10^4
10^{-5} M	6.1×10^4	4.3×10^5	2.8×10^4	2.1×10^5	2.8×10^5	1.6×10^6	6.5×10^4	3.9×10^5
