# Supplementary material for: Polycaprolactone-Based, Porous CaCO3 and Ag Nanoparticle Modified Scaffolds as a SERS Platform With Molecule-Specific Adsorption
Source: Front Chem. 2020 Jan 10;7:888. doi: 10.3389/fchem.2019.00888 (PMC6967418; doi:10.3389/fchem.2019.00888)
Supplement: Supplementary file 2 [file Data_Sheet_1.pdf]

Support information

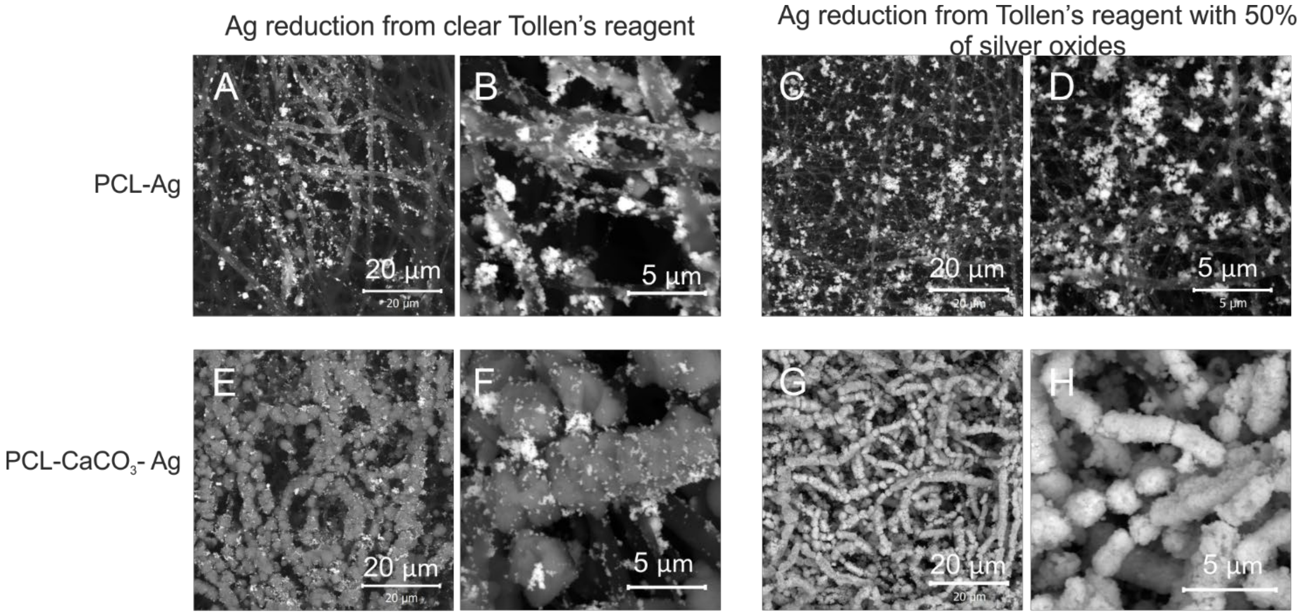

Figure S1. Scanning electron microscopy images.

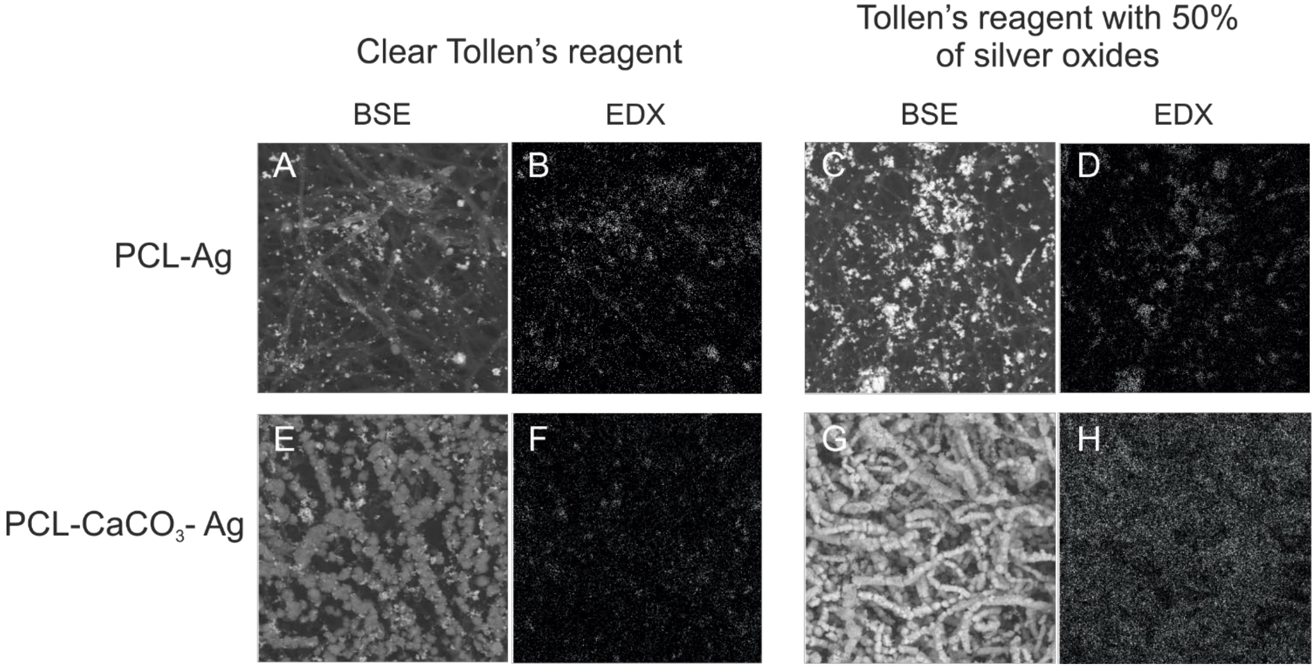

Figure S2. Electronic microscopy in back scattering electron mode (BSE) (A,C,E,G) and energe dispersive (EDX) (B,F,D,H) images of Ag ions distribution images of the PCL-Ag (A,B,C,D) and PCL-$CaCO_3$-Ag scaffold ((E,F,G,H) for silver reduction from the tollens reagent (A,B,E,F) and tollens reagent with 50% of the silver oxide.(C,D,G,H).

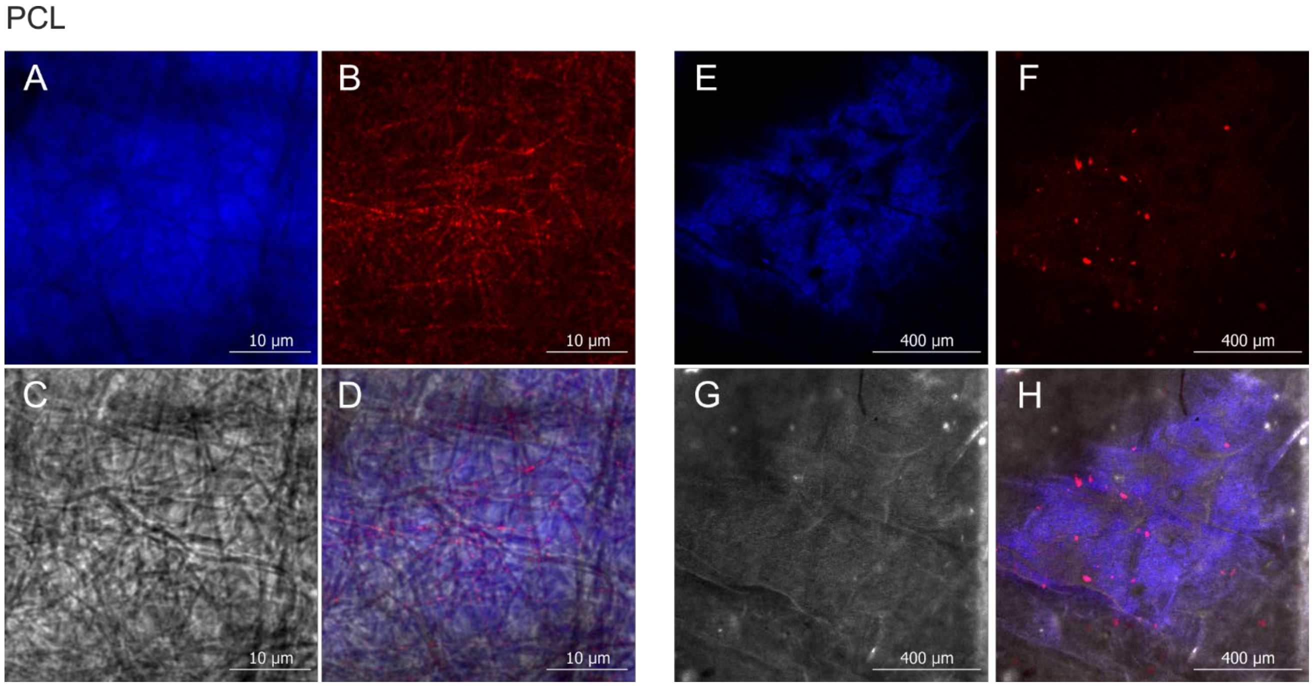

Figure S3. Confocal images of the PCL scaffold after incubation on mixture of Photosens and TRIC-BSA. A,E photosens channel, B,F TRITC-BSA channel, C,G the transmission channel. D,H overlayed channels.

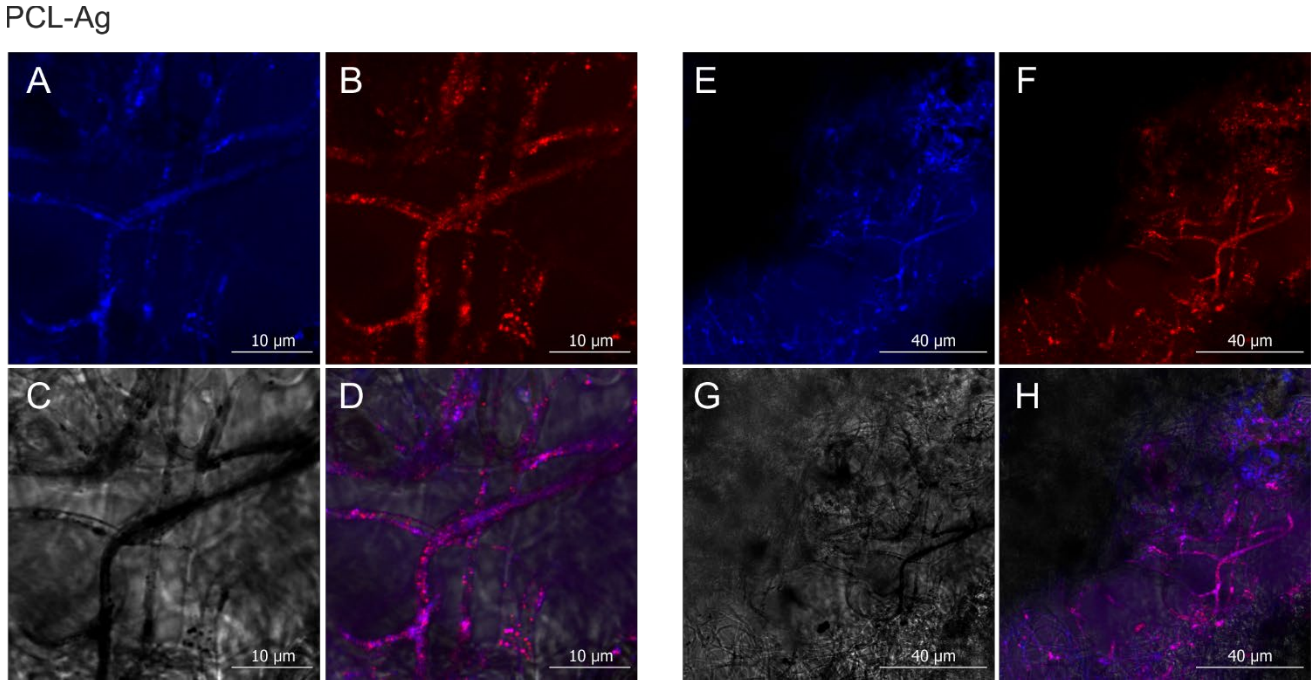

Figure S4. Confocal images of the PCL-Ag scaffold after incubation on mixture of Photosens and TRIC-BSA. A,E the blue channel for visualization Photosens molecules, B,F the red channel to visualize TRITC-BSA, C,G the transmission channel. D,H overlayed channels.

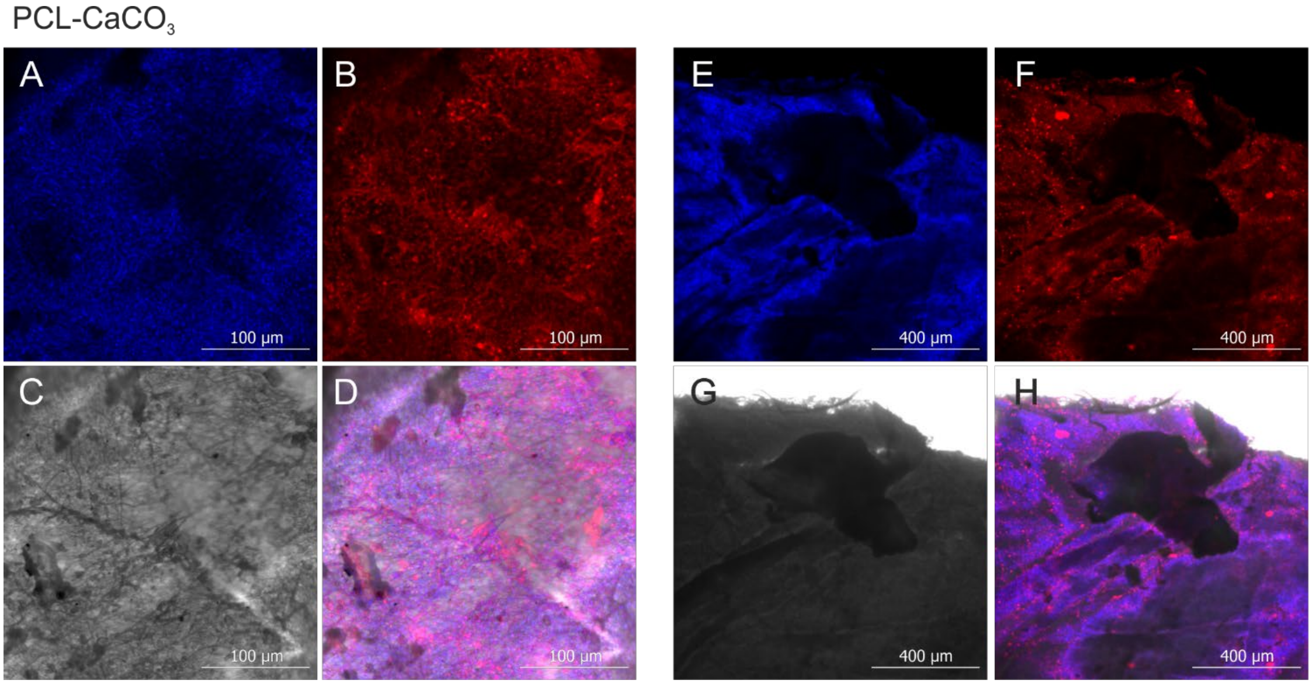

Figure S5. Confocal images of the PCL-$CaCO_3$ scaffold after incubation on mixture of Photosens and TRIC-BSA. A,E the blue channel for visualization Photosens molecules, B,F – the red channel to visualize TRITC-BSA, C,G the transmission channel. D,H overlayed channels.

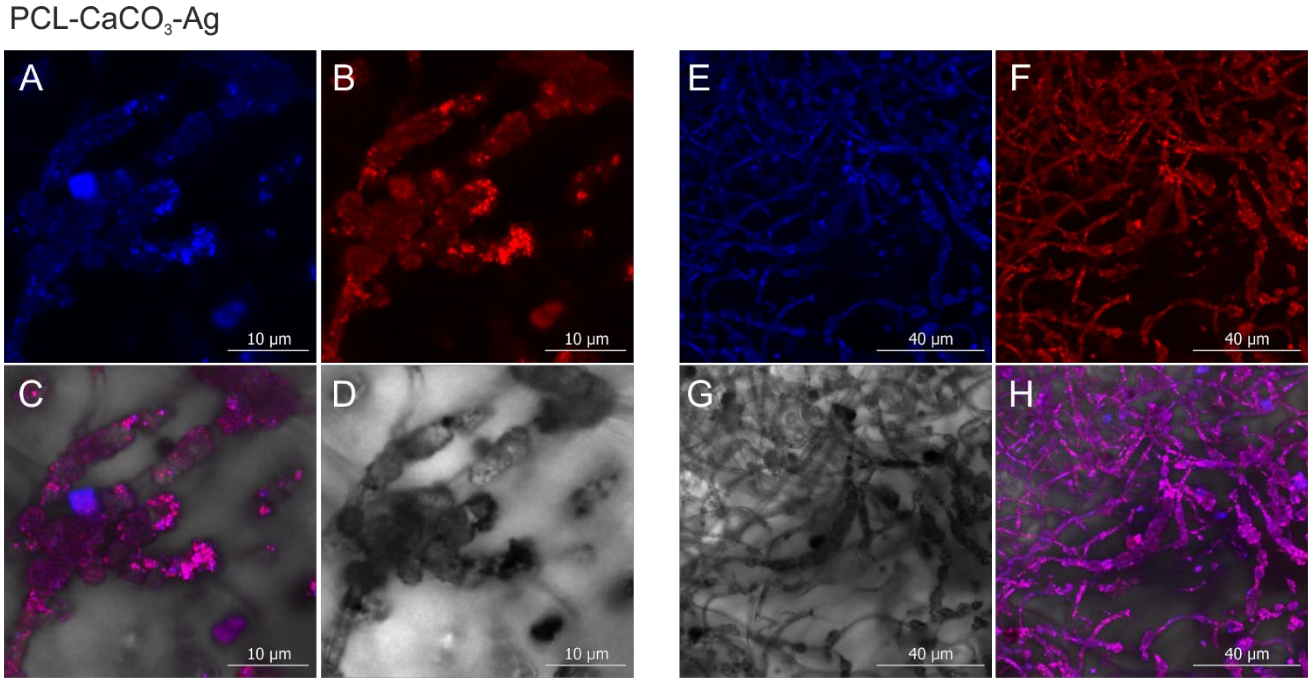

Figure S6. Confocal images of the PCL-Ag scaffold after incubation on mixture of Photosens and TRIC-BSA. A,E – the blue channel for visualization of Photosens molecules, B,F – the red channel to visualize TRITC-BSA, C,G the transmission channel. D,H overlayed channels.
